# The Role of AKR1B10 in Physiology and Pathophysiology

**DOI:** 10.3390/metabo11060332

**Published:** 2021-05-21

**Authors:** Satoshi Endo, Toshiyuki Matsunaga, Toru Nishinaka

**Affiliations:** 1 Laboratory of Biochemistry, Gifu Pharmaceutical University, Gifu 501-1196, Japan; 2 Education Center of Green Pharmaceutical Sciences, Gifu Pharmaceutical University, Gifu 502-8585, Japan; matsunagat@gifu-pu.ac.jp; 3 Laboratory of Biochemistry, Faculty of Pharmacy, Osaka Ohtani University, Tondabayashi 584-8540, Osaka, Japan; nisinat@osaka-ohtani.ac.jp

**Keywords:** aldo-keto reductases, AKR1B10, biomarkers

## Abstract

AKR1B10 is a human nicotinamide adenine dinucleotide phosphate (NADPH)-dependent reductase belonging to the aldo-keto reductase (AKR) 1B subfamily. It catalyzes the reduction of aldehydes, some ketones and quinones, and interacts with acetyl-CoA carboxylase and heat shock protein 90α. The enzyme is highly expressed in epithelial cells of the stomach and intestine, but down-regulated in gastrointestinal cancers and inflammatory bowel diseases. In contrast, AKR1B10 expression is low in other tissues, where the enzyme is upregulated in cancers, as well as in non-alcoholic fatty liver disease and several skin diseases. In addition, the enzyme’s expression is elevated in cancer cells resistant to clinical anti-cancer drugs. Thus, growing evidence supports AKR1B10 as a potential target for diagnosing and treating these diseases. Herein, we reviewed the literature on the roles of AKR1B10 in a healthy gastrointestinal tract, the development and progression of cancers and acquired chemoresistance, in addition to its gene regulation, functions, and inhibitors.

## 1. Introduction

Aldo-keto reductases (AKRs) are a group of NAD(P)(H)-dependent enzymes catalyzing interconversions between the carbonyl and alcohol groups of endogenous and xenobiotic compounds [1]. The AKR superfamily is systematized into 16 families: AKR1 (aldehyde reductases, aldose reductases, hydroxysteroid dehydrogenases, and steroid 5b-reductases); AKR2 (mannose and xylose reductases); AKR3 (yeast AKRs); AKR4 (chalcone and codeinone reductases); AKR5 (gluconic acid reductases); AKR6 (β-subunits of the potassiumgated voltage channels); AKR7 (aflatoxin dialdehyde and succinic semialdehyde reductases); AKR8 (pyridoxal reductases); AKR9 (aryl alcohol dehydrogenases); AKR10 (*Streptomyces* AKRs); AKR11 (*Bacillus* AKRs); AKR12 (*Streptomyces* sugar aldehyde reductases); AKR13 (hyperthermophilic bacteria reductases); AKR14 (*Escherichia coli* reductases), AKR15 (*Mycobacterium* reductases), and AKR16 (*Vibrio cholerae* reductases). Each family is further divided into several subfamilies based on a >60% amino acid sequence identity. To date, fifteen AKR members have been identified in humans and belong to the AKR1A, AKR1B, AKR1C, AKR1E, AKR6A, and AKR7A subfamilies. There are three members of the human AKR1B subfamily: AKR1B1 (aldose reductase), AKR1B10 (aldose reductase-like protein-1), and AKR1B15, whose genes are clustered at chromosome 7q33 [1]. AKR1B1, AKR1B10, and an enzymatically active isoform of AKR1B15 are 36-kDa soluble monomeric proteins consisting of 316 amino acids and sharing >68% amino acid sequence identity, of which 91.5% are shared between AKR1B10 and AKR1B15 [2,3]. The three AKRs are NADPH-dependent reductases and display overlapping substrate specificities for aromatic and aliphatic aldehydes but differ in their catalytic efficiencies [2,3,4,5,6], which is notably higher for retinal (all-*trans*-retinaldehyde) in AKR1B10 [5]. In addition, the glucose reductase activity characteristics of AKR1B1 are very low for AKR1B10 and AKR1B15 [2,4,5], and prostaglandin F synthase activity is observed with AKR1B1, but not with AKR1B10 [7]. In contrast to AKR1B1, AKR1B10 and AKR1B15 exhibit low 17β-hydroxysteroid dehydrogenase activity for estrone and 4-androstene-3,17-dione [3,6]. For subcellular localization, AKR1B1 and AKR1B10 are cytosolic, whereas AKR1B15 is in the mitochondria [3]. The three AKR1Bs also have different tissue distributions. While AKR1B1 is ubiquitous, AKR1B10 protein is predominantly expressed in the human stomach and intestine [2,8], although its mRNA is detected in many other tissues [3,8,9]. The mRNA for AKR1B15 is predominantly expressed in the placenta, testis, skeletal muscle, and adipose tissue, where its level is lower than that of mRNA for AKR1B10 [3].

While the most studied enzyme of the AKR1B subfamily is AKR1B1, which has been implicated in the pathogenesis of diabetes complications and inflammatory disease [10,11], many studies of AKR1B10 have focused on its association with cancers and other diseases since its overexpression in hepatocellular carcinomas (HCC) was found in 1998 [12]. In addition to its role in gastrointestinal homeostasis, increasing evidence suggests that the aberrant expression of AKR1B10 promotes its diagnostic and prognostic utility as a potential tumor biomarker and elucidates its role in carcinogenesis, tumor progression, and the development of chemoresistance. In this review, we summarize recent progress towards understanding the gene regulation of AKR1B10 and its functions in gastrointestinal physiology, the pathogenesis of several cancers and skin diseases, and acquired drug resistance. Based on the available evidence, we propose that AKR1B10 is thus a potential target for drug discovery. We also provide a brief overview of AKR1B10 inhibitors.

## 2. Gene Regulation of AKR1B10

### 2.1. Factors Regulating AKR1B10 Expression

AKR1B10 is induced in various types of cancer tissues and down-regulated in gastrointestinal cancers. In addition to tumor progression, changes in AKR1B10 expression levels are associated with several noncanceraous diseases and chemoresistance (as described later). Thus, AKR1B10 expression is an important factor in the pathogenesis of these diseases. However, the molecular mechanisms of AKR1B10 gene regulation have not been fully elucidated.

**Figure 1:**
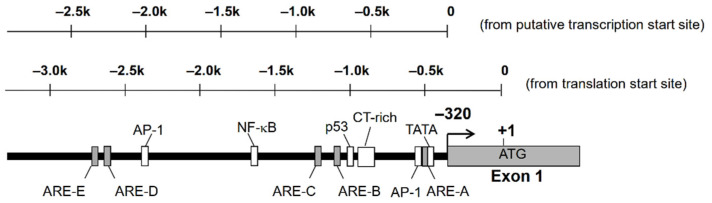
Putative transcription factor binding sites on the 5′-flanking region of AKR1B10 gene.

Nucleotide sequence analysis of the 5′-flanking region of AKR1B10 gene revealed the existence of putative TATA box, CAAT box, p53, AP-1, and antioxidant response elements (ARE) [13,14]. There is a complex microsatellite composed of repetitive C and T sequences, which are highly polymorphic and may affect the expression of AKR1B10 (Figure 1). We found polymorphism at this microsatellite in the human lung adenocarcinoma A549 cell line, but no significant difference in promoter activity was observed in the gene reporter assay analysis [14].

AKR1B10 expression is affected by treatment of human cultured cells with various agents (Table 1). Most of the agents upregulate AKR1B10, whereas a phorbol ester 12-*O*-tetradecanoylphorbol 13-acetate (TPA) down-regulates the expression [15]. 5-Fluorouracil (5-FU) and oxaliplatin (L-OHP) exhibit opposite effects on the AKR1B10 expression depending on colorectal cancer (CRC) cell lines [16]. Regulators and signal molecules involved in the modulation of AKR1B10 expression by several agents are also listed in Table 1.

### 2.2. Contribution of Nrf2 to AKR1B10 Induction

An antioxidant (ethoxyquin) induces AKR1B10 expression in lung cancer cell lines A549 and H23 [14]. Ethoxyquin is known to activate the transcription factor Nrf2 (NF-E2-related factor). Furthermore, increased AKR1B10 promoter activity caused by ethoxyquin was suppressed by the introduction of dominant-negative Nrf2 protein in the gene reporter assay. In addition, the introduction of Nrf2 augmented promoter activity, indicating that AKR1B10 gene transcription is regulated by Nrf2. Nrf2 is a member of the Cap‘n’Collar family of basic leucine zipper transcription factors known to be activated by various antioxidants or reactive oxygen species (ROS). Nrf2 forms a heterodimer with small Maf proteins and binds to the ARE of target genes [24,25]. In non-stimulated cells, Nrf2 is trapped, leading to ubiquitin-dependent degradation by Kelch-like ECH-associated protein 1 (Keap1). Once the cells are stimulated, the Keap1 protein is oxidatively modified and releases Nrf2, allowing its nuclear localization [26,27]. It has been suggested that Nrf2 is involved in the up-regulation of AKR1B10 by proteasome inhibitors [17], 9,10-phenanthrenequinone [22], and doxorubicin [18]. In addition, microarray analyses consistently indicate that the Keap1/Nrf2 pathway is involved in AKR1B10 gene regulation [28,29,30]. We demonstrated in the gene reporter assay that the introduction of dominant-negative Nrf2 protein also leads to a reduction in basal transcriptional activity of AKR1B10 in A549 cells, where AKR1B10 is highly expressed. However, the treatment failed to show any effect in H23 cells where AKR1B10 expression was very low, suggesting that Nrf2 participates in the basal expression of AKR1B10 [31]. Thus, the Keap1/Nrf2 pathway is one of the major regulatory systems for AKR1B10 gene regulation.

**Figure 2:**
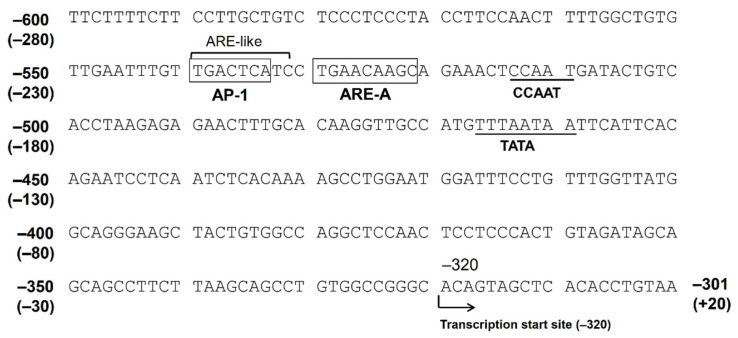
The nucleotide sequence of the 5′-flanking region of the AKR1B10 gene. The numbers represent the nucleotide position when +1 is defined as the translation start site. The numbers in parentheses represent the position when +1 is defined as the putative transcription start site.

The Nrf2 ARE consensus sequence is 5′-TGACnnnGC-3′, and there are at least five potential AREs found in the 5′-flanking region, up to −3282 bp from the translation start site (−2962 bp from the putative transcription start site). We indicated that only AREs located between −530 bp and −522 bp (between −210 bp and −202 bp from the transcription start site, ARE-A) are functional among these five AREs [31]. There is an AP-1 site (between −540 bp and −532 bp) just upstream of this ARE. The consensus sequence of AP-1 is 5′-TGACTCA-3′, and the corresponding nucleotide sequence of the AKR1B10 gene, 5′-TGACTCATC-3′, resembles the ARE. In fact, the introduction of a mutation in the AP-1 site resulted in a reduced response to Nrf2 in the gene reporter assay. Accordingly, this AP-1 site may function as an ARE, and this tandem repeat of AREs may be important for a strong response to stimulations targeting Nrf2 [31] (Figure 2). 

### 2.3. The Function of AP-1 Protein in AKR1B10 Gene Regulation

The induction of AKR1B10 by EGF and insulin in HepG2 cells also shows the importance of the AP-1 site [19]. The AP-1 protein consists of a heterodimer of c-Jun and c-Fos proteins binding to the AP-1 site [32]. Cell treatment with EGF or insulin significantly increases the amount of AP-1 protein. The introduction of c-Jun and c-Fos proteins also augments transcriptional activity in gene reporter assays. Interestingly, the injection of c-Fos shRNA to HepG2-inoculated mice to knockdown c-Fos results in the loss of AKR1B10 expression, suggesting that endogenously expressing c-Fos is an important regulator in the expression of AKR1B10. Furthermore, Cheng et al. [33] have proposed that AP-1 is involved in the induction of AKR1B10 in HCC cells. They show that the knockdown of interleukin-1 associated kinase 1 (IRAK1) leads to a decrease in AKR1B10 expression possibly mediated through regulating AP-1 signal transduction. 

On the other hand, the c-Jun component of AP-1 protein has the opposite function on AKR1B10 gene regulation. We have shown that TPA downregulates AKR1B10 expression through the induction of c-Jun protein in A549 cells [15]. In addition, the forced expression of c-Jun protein in A549 cells resulted in decreased AKR1B10 expression, and the introduction of c-Jun in gene reporter assays led to the suppression of transcriptional activity in an AP-1 site-independent manner. The leucine zipper domain is necessary for c-Jun to exhibit its suppressive action, indicating that c-Jun needs to interact with other proteins. Hence, c-Jun also acts as a negative regulator. Although it is not clear how c-Jun acts differently, the variations of other protein factors existing in the cells, such as c-Fos protein, may determine the action of c-Jun.

### 2.4. Signal Transduction

The cAMP-dependent signaling pathway may take part in the regulation of AKR1B10, because a phosphodiesterase inhibitor, IBMX, with BMP2, is able to induce AKR1B10 expression [21]. Since a potential cAMP-response element (CRE) has not been identified in the AKR1B10 gene promoter region to date, it could be that this pathway might modify the other signaling cascades. Extracellular signal-regulated kinase (ERK), a mitogen-activated protein kinase (MAPK), is an important signal molecule in AKR1B10 gene regulation. The induction of AKR1B10 by EGF and insulin is mediated through the activation of ERK in HepG2 cells, and totally abolished by the inhibition of its upstream kinase, MAPK/ERK kinase (MEK) [19]. Thus, the receptor tyrosine kinase/ERK cascade is one of the primary signaling pathways involved in the induction of AKR1B10. In addition, ERK regulates the basal expression of AKR1B10 in this cell line as well as in A549 cells, which is suggested by studies using MEK inhibition [15]. On the other hand, ERK activation is also a key event in the TPA-mediated down-regulation of AKR1B10. The inhibition of MEK resulted in decreased c-Jun induction, leading to the loss of TPA’s action. It is very interesting that ERK activation is involved in both up-regulation and down-regulation of AKR1B10 expression as seen in the case of c-Jun. 

Recently, p53 protein, a tumor suppressor gene product, was shown to play a key role in determining whether AKR1B10 expression is upregulated or down-regulated. In CRC HT29 cells possessing mutant p53, treatment with 5-FU or L-OHP reduced AKR1B10 expression. Alternatively, expression was induced by these agents in c CRC HCT116 cells having wild-type p53. When the p53 gene in HCT116 cells is knocked-down, 5-FU can no longer induce AKR1B10 [16]. Thus, p53 protein might act as a switch to determine the direction of AKR1B10 expression, suggesting that the level and status of p53 might be an important factor.

**Figure 3:**
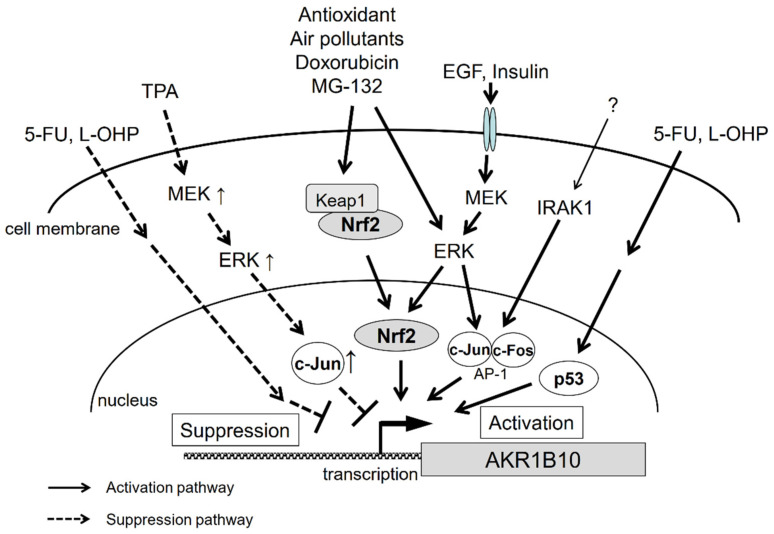
Signal transduction pathways that regulate AKR1B10 gene expression.

The gene regulation pathways for AKR1B10 are summarized in Figure 3. As described above, we still have little knowledge of the mechanisms underlying AKR1B10 gene regulation. The regulation pathways appear to be complex, since, for example, the same factors can produce opposing outcomes. There are likely more factors involved, and therefore, further investigations are necessary to solve the puzzle of gene regulation of AKR1B10 and its physiological significance.

## 3. AKR1B10 as a Multifunctional NADPH-Dependent Reductase

**Figure 4:**
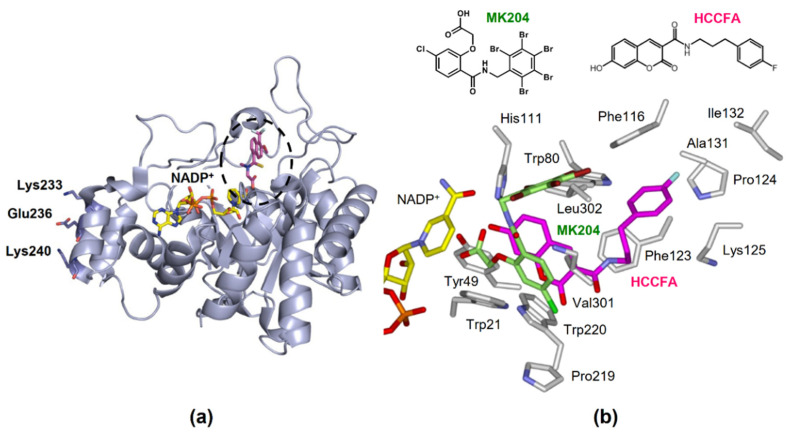
The structure of AKR1B10. (**a**) Tertiary structure complexed with NADP^+^ and an inhibitor tolrestat (PDB 1ZUA), showing the active site (dotted circle) and residues involved in binding to HSP90α. (**b**) The orientations of HCCFA (pink) and MK204 (light green) in the active site.

AKR1B10 reduces a variety of endogenous carbonyl compounds, such as retinals, lipid peroxidation-derived cytotoxic aldehydes, and isoprenyl aldehydes. It also metabolizes xenobiotics, including several drugs and polycyclic aromatic hydrocarbon (PAH) derivatives found in cigarette smoke and the environment. Like all AKR1 subfamily enzymes, AKR1B10 catalyzes a sequential-ordered Bi-Bi kinetic mechanism, with NADPH binding occurring before substrate binding [4]. The active site of AKR1B10 is located above its α/β-barrel tertiary structure consisting of eight α-helices and β-sheets (Figure 4a).

### 3.1. Retinoid Metabolism

**Figure 5:**
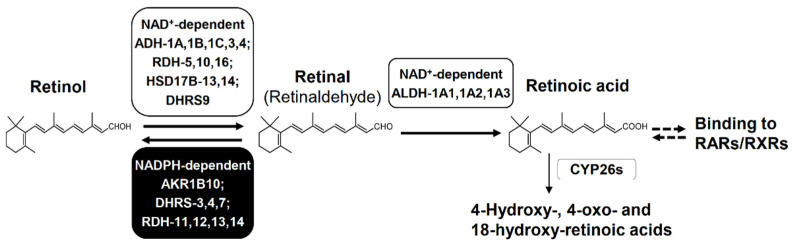
Biosynthesis and degradation of retinoic acid. Retinol is oxidized to retinal by NAD^+^-dependent enzymes, but this step is reversible by NADPH-dependent reductases. Retinal is oxidized irreversibly to retinoic acid by NAD^+^-dependent enzymes. Retinoic acid activates RAR and RXR to initiate the transcription of target genes that regulate differentiation, apoptosis, and cell cycle arrest. Retinoic acid is also eliminated by conversion to its hydroxy- and/or oxo-metabolites. Abbreviations: ADH, alcohol dehydrogenase; RDH, retinol dehydrogenase; HSD17B, 17β-hydroxysteroid dehydrogenase; DHRS, dehydrogenase/reductase (SDR family) member; ALDH, aldehyde dehydrogenase; CYP26s, cytochrome P450 family 26 enzymes, RAR, retinoic acid receptor; and RXR, retinoid X receptor. DHRS9 is called RDHL, and ALDH-1A1, 1A2, and 1A3 are also called retinal dehydrogenase (RALDH)-1, 2, and 3, respectively.

AKR1B10 efficiently catalyzes the reduction of retinal (i.e., all-*trans*-retinaldehyde) to retinol, which is the first reversible step of retinoid metabolism producing retinoic acid (i.e., all-*trans*-retinoic acid) that plays a pivotal role in proliferation, differentiation and morphogenesis of many cell types through binding to retinoic acid receptors or retinoid X receptors [34,35] (Figure 5). The reduction of retinal is mediated by other enzymes including AKRs (1B1, 1B15, 1C3 and 1C4), retinol dehydrogenases (RDHs) and dehydrogenase/reductase (SDR family) members (DHRSs). The *k*_cat_/*K*_m_ value for retinal (45 min^−1^ μM^−1^) of AKR1B10 is much higher than those (0.14–5.3 min^−1^ μM^−1^) of the other AKRs [5,34], DHRS4 [36], DHRS7 [37], and RDH13 [38], although its *K*_m_ value for retinal (0.6 μM) is higher than those of RDH11, RDH12 and RDH14 (0.04–0.15 μM) [38] and DHRS3 [39]. In addition, the *K*_m_ and *k*_cat_/*K*_m_ values of AKR1B10 are much lower and higher, respectively, than *K*_m_ (2.4–11 μM) and *k*_cat_/*K*_m_ values (1.6–7.6 min^−1^ μM^−1^) of aldehyde dehydrogenases (ALDHs: 1A1, 1A2 and 1A3) [40], which catalyze the oxidation of retinal to retinoic acid. Thus, AKR1B10 acts as one of the enzymes that control the cellular concentration of retinal, and contributes to maintain the retinoid homeostasis in normal gastrointestinal tissues with its high-expression. In extra-gastrointestinal tissues with little or no expression of AKR1B10, the retinoid metabolism proceeds normally by actions of other enzymes. However, the down-regulation of AKR1B10 in gastrointestinal cancers and its high expression in other cancers may disturb the retinoid homeostasis as described in Section 5 and Section 6.

### 3.2. Detoxification of Reactive Carbonyl Species (RCS)

The continuous oxidation of carbohydrates and lipids generates RCSs, such as methylglyoxal, 3-deoxyglucosone, hexanal, acrolein, 4-hydroxynonenal, 4-oxo-2-nonenal, and phospholipid aldehydes. These RCS are metabolized by several members of the AKR superfamily, as well as by ALDHs and alcohol dehydrogenases [41,42]. AKR1B10 reduces the above RCSs to their less-toxic alcohol [4,43,44,45,46,47,48], of which highly cytotoxic 4-hydroxynonenal and 4-oxo-2-nonenal are most efficiently reduced to their less-toxic alcohols by AKR1B10 among the members of the AKR superfamily. Kinetic constants for 4-hydroxynonenal are shown in Table 2, and AKR1B10 also exhibits high catalytic efficiency for 4-oxo-2-nonenal (*k*_cat_/*K*_m_ = 186 min^−1^ μM^−1^) [49]. Furthermore, the catalytic efficiency for 4-hydroxynonenal of AKR1B10 is higher than those of the ALDHs (1A1 and 1A3) that oxidize to 4-hydroxy-2-nonenoic acid (Table 2). Thus, AKR1B10 plays a key role in the detoxification of lipid peroxidation derived RCSs.

### 3.3. Isoprenoid Metabolism

AKR1B10 efficiently reduces farnesal and geranylgeranial into their alcohols (farnesol and geranylgeraniol) with low *K*_m_ (2.5 μM and 0.9 μM, respectively) and high *k*_cat_/*K*_m_ values (9.1 min^−1^ μM^−1^ and 8.3 min^−1^ μM^−1^, respectively) [4]. The catalytic efficiency of AKR1B10 is superior to other AKRs, although for geranylgeranial, it is similar to that of AKR1C3 [52]. Farnesol and geranylgeraniol are phosphorylated to their pyrophosphates, which are required for the transformational activity of many oncogenic proteins, including some RAS family members. The role of AKR1B10 in modulating the Kras-E-cadherin pathway is further supported by the overexpression, knockdown, and inhibition of the enzyme in pancreatic cancer CD18/HPAF and Panc10.05 cells and xenograft tumors [53,54]. It has also been suggested that the proliferative capacity of oxaliplatin-resistant CRC HT29 cells is affected by the modulation of isoprenoid metabolism by AKR1B10 [55].

### 3.4. Xenobiotic Metabolism

The substrate specificity of AKR1B10 is broad, reducing xenobiotic aromatic aldehydes [2,4], quinones [20,56], and ketones, including several drugs [57,58,59,60]. The drug substrates include dolasteron, oracin, daunorubicin [57], doxorubicin [58], nabumetone [59], and tiaprofenic acid [60], which are also reduced by other AKRs and carbonyl reductase 1. Among these drugs, dolasteron and nabumetone are efficiently reduced by AKR1B10, whose *k*_cat_/*K*_m_ values are higher than (or comparable to) those of the other reductases [57,59,61]. Since AKR1B10 is expressed abundantly in the intestine, it may play a significant role in the intestinal first-pass reductive metabolism of orally administered dolasteron and nabumetone. In addition, AKR1B10 reduces redox-active 9,10-phenanthrenequinone in diesel exhaust [20] and PAH *o*-quinones derived from the oxidation of PAH *trans*-dihydrodiols by the enzyme [62]. Furthermore, the gut microbiota produces various carbonyl compounds [63], several of which are reduced by AKR1B10 [2,4,5,43,44,45,47].

## 4. Moonlighting Functions of AKR1B10

AKR1B10 has three moonlighting functions that are independent of its enzyme activity.

(1) Fatty acid/lipid synthesis. In breast cancer RAO-3 cells, AKR1B10 interacts with acetyl-CoA carboxylase-α (a rate-limiting enzyme of de novo fatty acid synthesis), preventing acetyl-CoA carboxylase-α ubiquitination and proteolysis, and thereby promoting fatty acid/lipid synthesis [64].
(2) Interaction with heat shock protein (HSP) 90α in AKR1B10 secretion. Cytosolic AKR1B10 is secreted from cells through a lysosome-mediated nonclassical pathway, increasing its presence in breast cancer patients’ serum [65]. The AKR1B10 secretion is mediated by interaction with HSP90α, which binds to Lys-233, Glu-236, and Lys-240 in AKR1B10 [66] (Figure 4a).
(3) Interaction with glyceraldehyde-3-phosphate dehydrogenase (GAPDH). A recent report shows that AKR1B10 interacts with GAPDH in CRC HT29 cells [67]. The interaction inhibits the nuclear import of GAPDH, and subsequently results in autophagy repression, for which AKR1B10 reductase activity is likely to be important.

## 5. AKR1B10 in the Gastrointestinal Tract and Cancer

AKR1B10 is abundant primarily in human stomach, small intestine, and colon [2,8]. Immunohistochemical analysis has revealed that AKR1B10 is localized in the rapidly renewing epithelial cells of the colon [68] and stomach [69]. In contrast, AKR1B10 is markedly decreased or undetectable in cancerous lesions of the colon [68,70,71,72,73,74,75,76] and stomach [69,77,78,79] (Table 3), as well as in precancerous lesions of the colon (including ulcerative colitis), Crown’s disease, and adenomatous polyps [68,73]. Zu et al. [73] reported that AKR1B10 is critical for protecting host cells from DNA damage induced by electrophilic carbonyl compounds, which are ingested and/or metabolically formed in the intestine. The authors suggest the enzymatic activity (i.e., detoxification of RCS) of AKR1B10 is more important than its promotion of fatty acid synthesis for maintaining homeostasis of colon epithelium. However, whether other AKR1B10 functions (as described in Section 4) are involved in intestinal physiology remains to be elucidated. Additionally, since it activates ERK signaling (as described in Section 6), AKR1B10 might participate in the renewal of epithelial cells through the mitogen-activated protein kinase (MAPK) pathway that regulates the proliferation and differentiation of gastrointestinal epithelial cells [80]. 

The expression of AKR1B10 is decreased in patients with CRC and the low expression correlates with poor prognosis and reduced survival [16,71,74,75,76]. It has been suggested that AKR1B10 down-regulation results from mutation of the tumor suppressor p53 gene: Wild-type p53 is a transcriptional activator of AKR1B10, but mutant p53 acts as a repressor [16]. Recently, Li et al. [67] reported that AKR1B10 expression is negatively regulated by a membrane receptor neuropilin1 (NRP1), which was upregulated in CRC HT29 cells that underwent autophagy following glucose deprivation. This study also showed that AKR1B10 inhibits glucose-deprivation autophagy by interacting with GAPDH, and suggested that low AKR1B10 expression in CRC promotes tumor development by upregulated autophagy. 

Several studies suggest that decreased AKR1B10 expression mediates tumorigenesis. Ohashi et al. [71] reported that the decreased expression of AKR1B10 disrupts the tumor-suppressive function of p53. Kropotova et al. [72] observed a decrease in the expression of mRNAs for the retinoid metabolizing enzymes (AKR1B10, ADH1B, ADH1C, DHRS9, RDH5, and ALDH1A), and suggested that the resultant disturbance to retinoid homeostasis contributes to the progression of CRC. Zu et al. [73] suggest that AKR1B10 deficiency may leave the colonic HCT-8 cells vulnerable to electrophilic carbonyl lesions, leading to DNA damage and carcinogenesis. Recently, Yao et al. [75] reported that AKR1B10 depletion promotes the progression of CRC by down-regulating fibroblast growth factor (FGF)-1, which is related to the growth and migration of CRC and gastric cancer [100,101,102]. This finding implies that FGF-1 expression is low in a normal colon with high levels of AKR1B10, in contrast to the high levels of FGF-1 due to low AKR1B10 expression in CRC. Further studies of the underlying mechanism by which FGF-1 is suppressed by AKR1B10 might lead to the identification of a novel moonlighting function of AKR1B10. 

A poor prognosis in gastric cancer is suggested by the down-regulation of AKR1B10 expression [78]. By contrast, patients who underwent neoadjuvant chemotherapy showed that high AKR1B10 expression was associated with lymph node metastasis and a poorer prognosis, along with a weak response to neoadjuvant chemotherapy [79]. In addition, the overexpression of AKR1B10 in gastric cancer MKN46 cells stimulates migration [18] and down-regulates peroxisome proliferator-activated receptor-γ (PPARγ) that is closely linked to growth suppression and death of cancer cells [103]. However, there has been no mechanistic study relating AKR1B10 with gastric cancer progression, with the exception of Kropotova et al. [77], who suggested that retinoic acid synthesis was dysregulated due to significant decreases in the mRNAs for AKR1B10 and other retinoid-metabolizing enzymes (ADH4, ADH1B, ADH1C, DHRS9, and RDH12). Further studies to elucidate whether the low expression of AKR1B10 is associated with carcinogenesis are warranted.

## 6. Diseases Associated with AKR1B10 Elevation

### 6.1. Hepatocellular Carcinoma (HCC)

The overexpression of AKR1B10 in HCC was first identified by Scuric et al. [12] in 1998, which alluded to a role of AKR1B10 in liver carcinogenesis. Subsequent literature (through 2019) supporting the diagnostic and prognostic utility of AKR1B10 as a potential biomarker for HCC has been reviewed by DiStefano and Davis [104]. They summarize the roles of the enzyme in HCC development and progression through lipogenesis, oxidative stress, detoxification of cytotoxic RCS, and the regulation of sphingosine-1 phosphate and retinoic acid. Although this review also includes the underlying upregulation mechanisms of AKR1B10 in the process of hepatocarcinogenesis by microRNA (miR-383-5p), 14-3-3ε, and AP1 downstream of IRAK1 or EGFR signaling, a recent study reported that AKR1B10 elevation results from compensatory upregulation to protect hepatocytes against oxidative stress during hepatocarcinogenesis [105]. Thus, AKR1B10 is emerging as a promising biomarker for HCC. In addition to AKR1B10, HSP90α is also a potential serum biomarker for diagnosing α-fetoprotein negative HCC [106], as it interacts with AKR1B10 [66].

### 6.2. Nonalcoholic Fatty Liver Disease (NAFLD)

NAFLD encompasses a spectrum of liver pathologies which involve an accumulation of triglycerides in the hepatocytes, hepatocyte apoptosis, liver inflammation and fibrosis termed as non-alcoholic steatohepatitis (NASH), and, in extreme cases, it can progress to cirrhosis and HCC [107]. Since a significant increase of AKR1B10 in steatohepatitis was first found in 2012 [108], it has been confirmed in biopsy samples and sera from NAFLD patients [109,110,111,112,113,114,115,116], indicating that AKR1B10 is a consistent marker for the progression of NAFLD/NASH. Oxidative and endoplasmic reticulum (ER) stresses mediated by chronic lipotoxicity play a major role in the cellular mechanisms responsible for NAFLD/NASH pathogenesis [107] and induces Nrf2 transcriptional activity [117,118]. The upregulation of the Nrf2-target AKR1B10 gene in NAFLD is, thus, due to oxidative and ER stresses [115]. Furthermore, it may also be due to a reduction in pregnane X receptor (PXR) that negatively regulates the enzyme’s expression [110]. Several roles have been proposed for the upregulated AKR1B10 in NAFLD/NASH progression. This enzyme contributes to lipogenesis through the stabilization of acetyl-CoA carboxylase [110], in addition to detoxification of RCS derived from lipid peroxidation. This AKR1B10 overexpression, together with the underexpression of ALDHs (1A2 and 1A3) [111] and HSD17B14 [115], may reduce hepatic retinal levels, and hence lead to a decrease in retinoic acid, favoring progression of NASH to HCC. Furthermore, the immune deregulations associated with the progression of NAFLD may be related to AKR1B10 [116]. Two studies [113,115] suggest that AKR1B10 is a useful serum biomarker for advanced liver fibrosis and, thereby, the diagnosis of NAFLD/NASH. These studies recommend combining AKR1B10 with other markers such as Wisteria floribunda agglutinin-positive Mac-2-binding protein (WFA+-M2BP) [112] and growth/differentiation factor 15 (GDF15) [115].

### 6.3. Lung Cancer

There are two major types of lung cancer: small cell lung carcinoma and non-small cell lung carcinoma (NSCLC). Subclassifications of NSCLC include squamous cell carcinoma (SCC), adenocarcinoma (ADC), and large cell carcinoma. In 2005, Fukumoto et al. [8] were the first to report the overexpression of AKR1B10 in SCC and ADC. They proposed that AKR1B10 could be a potential diagnostic marker specific to NSCLC in smokers. The overexpression of AKR1B10 in NSCLC at the mRNA and/or protein level has been observed in many studies [81,82,83,84,85,86,87,88,89,90] (Table 3), which have shown SCC to have higher AKR1B10 expression than ADC [8,81,84,87]. Higher AKR1B10 expression may be due to the Nrf2 mutations that occur primarily in SCC [84]. The overexpression of AKR1B10 is a prognostic factor for NSCLC [82,89] and an indicator for poor recurrence-free survival in patients with resected ADC [88]. The upregulation of AKR1B10 may be involved in the carcinogenicity and metastasis of NSCLC by activating the ERK signaling pathway [87,89,90], as well as by the detoxification of RCS derived from lipid peroxidation and suppressing the synthesis of retinoic acid [85].

Similar to HCC and NAFLD, the oxidative stress-dependent activation of Nrf2 in NSCLC cells is a major factor responsible for upregulating AKR1B10 expression. However, a recent study with lung ADC A549 cells suggests another mechanism of AKR1B10 upregulation via a long non-coding RNA linc00665, which exerts its oncogenic role by acting as a competing endogenous RNA for miR-98 and subsequently increasing enzyme expression [89]. In addition, comparative analyses of lung specimens between healthy smokers and nonsmokers, and between smoking and non-smoking NSCLC patients, have shown that smoking mediates the upregulation of AKR1B10 expression [8,23,82,83,87,119]. AKR1B10 is also frequently overexpressed in interstitial pneumonia, a disease that increases the risk of lung cancer in smokers [120]. Thus, AKR1B10 is a diagnostic marker of NSCLC in smokers, as its smoking-induced upregulation may be an early indicator among the multiple events leading to lung cancer. AKR1B10 metabolizes PAH *trans*-dihydrodiols to redox-active *o*-quinones, enhancing the production of reactive oxygen species (ROS) and leading to further overexpression of Nrf2-target genes, including AKR1B10 [62]. Indeed, a redox-active PAH *o*-quinone, 9,10-phenanthrenequinone, is reduced by AKR1B10. Its treatment of A549 cells induces the expression of AKR1B10 through ROS- and Nrf2-dependent mechanisms, leading to further progression of malignant lung cancer cells through activating the ERK pathway [22]. Thus, AKR1B10 may be involved in the initial steps of carcinogenesis caused by smoking.

### 6.4. Breast Cancer

Breast cancer RAO-3 cells exhibit elevated AKR1B10 expression [64], and the high expression is observed in situ and in the serum of the patients, suggesting that AKR1B10 expression may be a diagnostic and therapeutic marker [91,92,93,94,95] as well as a poor prognostic factor for breast cancer [91,95] (Table 3). Notably, AKR1B10 expression is significant in breast cancers that are positive for human epidermal growth-factor receptor type 2 (HER2) [92,96]. Several roles have been proposed for AKR1B10 in cancer growth and metastasis. AKR1B10 may support breast cancer cell survival by releasing carbonyl stress and promoting fatty acid/lipid synthesis [91]. In addition, AKR1B10 promotes cancer metastasis through activation of the ERK signaling pathway, which stimulates the downstream integrin α5/δ-catenin mediated FAK/Src/Rac1 signaling pathway [93] and increases the expressions of matrix metalloproteinase-2 (MMP2) and vimentin [95]. The proposed mechanism underlying the activation of the ERK signaling pathway by AKR1B10 is as follows: AKR1B10 promotes lipogenesis, which leads to increases in second messengers (PIP_2_, DAG, and IP_3_) of the PKC-mediated ERK signaling cascade [121]. However, van Weverwijk et al. [96] show that lipogenesis is significantly reduced in breast cancer cells with high AKR1B10 expression. Instead, fatty acid oxidation is sustained by AKR1B10-mediated limitation of the toxic side effects of oxidative stress. Thus, further research on lipid metabolism in breast cancer is warranted.

### 6.5. Pancreatic Cancer

In pancreatic cancer, Keap1–Nrf2 signaling is dysregulated and Nrf2-ARE-driven genes are frequently upregulated [122]. As an Nrf2-ARE effector protein, AKR1B10 is overexpressed in pancreatic adenocarcinoma, intraepithelial neoplasia [54], and mucinous cystic pancreatic tumors [97] (Table 3). AKR1B10 efficiently reduces isoprenyl aldehydes (farnesal and geranylgeranial) to protein prenylation precursors (farnesol and geranylgeraniol) [52]. Mechanistic studies show that the upregulated enzyme promotes cancer cell growth by activating ERK signaling via modulating the prenylation of Kras protein [53,54].

### 6.6. Oral Cancer

More than 90% of oral cancers are squamous cell carcinomas (OSCC), in which AKR1B10 is overexpressed (Table 3). As such, AKR1B10 is a prognostic biomarker for predicting recurrence and survival in OSCC [98,99]. In addition, patient salivary levels of AKR1B10 are high and correlated with poor prognosis and elevated tumor recurrence incidence in OSCC [123]. AKR1B10 is thought to increase the prenylation of Ras protein that is critical in OSCC [98], as described in the pancreatic cancer section (Section 6.5). Several environmental factors increase the risk of OSCC, the greatest of which is cigarette smoking. Cigarette smoke condensate induces AKR1B10 expression in oral dysplasia (Leuk1 and Leuk2) and OSCC 101A cells [124], in which AKR1B10 may activate pro-carcinogen PAHs, promoting carcinogenesis, as described in Section 6.4.

### 6.7. Other Cancers

Uterine cancer. Immunohistochemical analysis has detected AKR1B10 expression in 20.0% and 15.8% of cervical and endometrial cancer cases, respectively. Tumor recurrence was associated with AKR1B10 expression after surgery for cervical cancer [125]. In subsequent studies on endometrial cancer [126,127], the expression of AKR1B10 at the mRNA level was significantly increased, while there were significantly decreased protein levels. Differences in the expression of AKR1B10 among these studies might be explained by the type of cancer cells, as cervical and endometrial cancers are squamous cell carcinomas and adenocarcinomas, respectively.

Renal cancer. In the Human Protein Atlas database (https://www.proteinatlas.org, [accessed on 2 February 2021]), AKR1B10 levels in renal cancer are significantly high and unfavorable for prognosis. There is only one report in the literature showing that AKR1B10 is highly expressed in specimens of hereditary and sporadic type 2 papillary renal cell carcinoma, in which Nrf2 is activated by fumarate hydratase mutations [128].

Nasopharyngeal carcinoma (NPC). AKR1B10 expression is increased in NPC tissues (compared to the normal tissues), in which squamous cell carcinomas show higher AKR1B10 expression than basal cell carcinomas, adenoid cystic carcinomas, adenocarcinomas, and undifferentiated carcinomas. However, AKR1B10 expression levels in NPC tissues are lower than those in nasopharyngeal hyperplasia and benign tumors, where AKR1B10 was predominantly present in nuclei [129]. Guo et al. also reported the lower expression of AKR1B10 in NPC than benign tumors, and they suggested that AKR1B10 may be involved in regulating the proliferation and migration of NPC cells [130]. In contrast, AKR1B10 was overexpressed in NPC tissues from patients resistant to radiotherapy, thereby suggesting that AKR1B10 confers radio-resistance [131]. 

Esophageal carcinoma. AKR1B10 expression is increased in epithelium specimens from patients with erosive gastro-esophageal reflux disease and Barrett’s esophagus but not in those with esophageal adenocarcinoma [132,133]. Since high expression of AKR1B10 has been observed in an esophageal squamous cell carcinoma cell line, OE-21 [132], it would be interesting to analyze the expression of AKR1B10 in specimens from patients with squamous cell carcinoma, which is the predominant form of esophageal carcinoma worldwide.

### 6.8. Non-Neoplastic Skin Diseases

The up-regulation of AKR1B10 has been observed in skin lesions of patients with psoriasis [134,135], keloids [136,137], atopic dermatitis [138], and type 2 reaction leprosy [139]. Psoriasis and keloid lesions show extremely high AKR1B10 expression levels (23-86-fold higher than normal skin) [134,135,136,137]. Although the role of AKR1B10 in the pathogenesis of atopic dermatitis and type 2 reaction leprosy remains unknown, its overexpression is related to the proliferation and migration of keratinocytes in psoriasis [135] and causes the dysregulation of retinoid metabolism, one of proposed mechanisms contributing to keloid disease [137,138]. The AKR1B10-overexpressing keratinocytes also secrete paracrine signals that enhance the fibrogenic activity of dermal fibroblasts and contribute to keloid pathobiology [136,137].

## 7. AKR1B10 in Anti-Cancer Drug Resistance

Chemotherapy is a common therapeutic approach for treating many cancers. However, primary or acquired drug resistance is a major problem during chemotherapy, which consequently leads to malignant transformation and metastasis. Human AKRs (1B1, 1B10, 1C1, 1C2, and 1C3) are regarded as novel factors in chemoresistance, along with drug transporters, growth factor receptors, and the ubiquitin-proteasome system [55,140,141]. Among the five AKRs, AKR1B10 may play a crucial role in acquiring resistance to several anti-cancer drugs. In clinical studies, bladder cancer specimens receiving carboplatin-gemcitabine combination chemotherapy displayed higher levels of AKR1B10 expression compared to the pre-chemotherapy group [142]. A study on patients with gastric cancer who underwent neoadjuvant chemotherapy showed that high AKR1B10 expression was associated with lymph node metastasis and a poor response to neoadjuvant chemotherapy [79]. These two studies also indicate that AKR1B10 upregulation is associated with a poorer prognosis for patients receiving chemotherapy. In addition, the upregulation of AKR1B10 has been observed in several cancer cell variants established by continuous exposure to anti-cancer drugs, as summarized in Table 4. In these resistant cells, chemoresistance was overcome by the addition of an AKR1B10 inhibitor or siRNA-mediated silencing [14,48,55,103,143,144,145]. Thus, AKR1B10 is considered a key factor responsible for the development of chemoresistance in these cancer cells.

The most accepted mechanism underlying the upregulation of AKR1B10 is the constitutive activation of Nrf2, a transcription factor involved in the gene expression of AKR1B10, as described in Section 2. Although specific details of the mechanism(s) of constitutive Nrf2 activation remain unclear, some researches propose an association between the development of chemoresistance and somatic mutations in Nrf2/Keap1, based on results showing a loss of the Nrf2-Keap1 interaction in cancer cell lines showing chemoresistance [148,149,150]. Another study also concluded that EGF induces AKR1B10 expression through AP-1 signaling in HCC HepG2 and Hep3B cells [18]. Moreover, recent transcriptome sequencing of HCC samples found a significant upregulation of IRAK1, which is likely to confer chemoresistance through a signaling pathway dependent on AP-1 and AKR1B10 [33]. Thus, AKR1B10 is considered to be upregulated through various processes, including Keap1 mutation, EGF exposure, and IRAK1 overexpression (Figure 3). Further studies are warranted to clarify the effect of AKR1B10 gene regulation on developing chemoresistance.

Studies with chemoresistant cells suggest that the role of AKR1B10 in chemoresistance development is dependent on the anti-cancer drugs or cancer cells used. While AKR1B10 may contribute to oxaliplatin-resistant HT29 cells through modulating isoprenoid metabolism [55], its role in chemoresistance to anthracycline drugs is performed by modulating the metabolism of the drugs to less toxic metabolites by reducing the C13 ketone group [58,146,151]. Exposing doxorubicin, docetaxel, cisplatin, and mitomycin c to resistant cancer cells generates ROS, which causes lipid peroxidation and subsequent increases in RCS, including highly cytotoxic 4-hydroxynonenal and 4-oxo-2-nonenal [18,48,55,143,144,145]. As described in Section 3.2, AKR1B10 efficiently detoxifies the two cytotoxic aldehydes. In addition, knockdown of the AKR1B10 gene by siRNAs sensitizes CRC HCT-8 cells to acrolein and crotonaldehyde [152]. Thus, the detoxification of RCS by AKR1B10 is the most critical step in developing resistance to these anti-cancer drugs. 

In the doxorubicin-resistant MKN45 cells, it has been suggested that AKR1B10 facilitates the induction and secretion of MMP2 through activation of the ERK signaling pathway and the above-mentioned isoprenoid metabolism with consequent activation of small-G protein [18]. In cisplatin-resistant MKN45 and LoVo cells, AKR1B10 is proposed to be involved in the down-regulation of PPARγ through activation of the isoprenoid-dependent signaling pathway and/or the catalysis of PPARγ substrates [103]. Further studies are needed to elucidate the detailed mechanism(s) underlying the induction of MMP2 and down-regulation of PPARγ.

## 8. AKR1B10 Inhibitors

Inhibitors of AKR1B10 are considered promising candidates for treating extra-gastrointestinal cancers and chemoresistance described in Section 6 and Section 7. Since aldose reductase (AKR1B1) has a similar structure to AKR1B10 and is involved in glucose and prostaglandin metabolism, developing inhibitors as anti-cancer drugs or adjuvant therapies for chemotherapeutic drug resistance must ensure selective inhibition of AKR1B10. Previously, Hung et al. [153] reviewed various synthetic and natural AKR1B10 inhibitors reported until 2015 [153]. Among them, HAHE [154] was the most potent, and oleanolic acid was the most selective to AKR1B10 compared to AKR1B1 [155]. Table 5 shows the inhibitory potency and selectivity (ratio of AKR1B10 to AKR1B1) of the inhibitors reported only from 2016 onwards, in addition to those of 3-(4-hydroxy-2-methoxyphenyl)acrylic acid 3-(3-hydroxyphenyl)propyl ester (HAHE), oleanolic acid, epalrestat, and glycyrrhetinic acid [156], which were used in studies on cellular AKR1B10-mediated metabolism. The natural inhibitors include flavonoid emodin [157], unsaturated fatty acid arachidonic acid [158], cohumulone, and 8-prenylnaringenin, the latter two of which are plant hop-derived α-bitter acid and prenylflavanone, respectively [159,160]. Although cohumulone is highly selective, emodin potently inhibits human carbonyl reductase 1 [157] with S-nitroso-glutathione reductase activity [161]. Furthermore, unsaturated fatty acids, including arachidonic acid, are similarly inhibitory to both carbonyl reductase 1 and human four AKRs (1C1-1C4) with hydroxysteroid dehydrogenase activity [162,163]. In contrast, the synthetic inhibitors, 7-Hydroxy-2-oxo-2*H*-chromene-3-carboxylic Acid [3-(4-Fluorophenyl)propyl]amide (HCCFA) [164], MK204 [165], and androst-3β,5α,6β,19-tetrol [166], show greater inhibitory potency and/or selectivity. HCCFA is the most potent and shows negligible or low inhibition to human carbonyl reductase 1 and AKRs (1A1, 1C1, 1C2, 1C3, and 1C4). HCCFA has greater chemical stability than HAHE. The inhibitors bind to the active site of AKR1B10 [164,165], in which their hydroxyl or carbonyl group interacts with the catalytically important residues, Tyr49 and His118. However, the orientations of their other parts are different, reflecting their chemical structures (Figure 4b). The residue differences between AKR1B10 and AKR1B1 at positions 114, 125, 301, and/or 304 may contribute to selective inhibition [154,155,164,165].

Among the above inhibitors, only HCCFA significantly suppresses not only migration, proliferation and metastasis of lung cancer A549 cells, but also metastatic and invasive potentials of cisplatin-resistant A549 cells [164]. Of the previously discovered inhibitors, HAHE and oleanolic acid suppress the proliferative potential of gastric carcinoma MNK45 and CRC Lovo cells that are resistant to cisplatin [103] and doxorubicin [144]. Oleanolic acid also increases the drug sensitivity of docetaxel-resistant A549 and prostate cancer Du145 cells [145]. Although epalrestat may be harmful to CRC HCT-8 cells by promoting DNA damage [73], it enhances the efficacy of sorafenib (an anti-cancer drug) on HCC by inhibiting AKR1B10 [167]. In addition, glycyrrhetinic acid may attenuate disturbed vitamin A metabolism in NAFLD and/or NASH through AKR1B10 inhibition [168]. Since HCCFA, MK204, and oleanolic acid are commercially available, they can be used for elucidating the mechanisms underlying tumorigenesis of AKR1B10-overexpressing cancers and chemotherapy resistance, which would precede AKR1B10-based therapies in clinical cancer management.

## 9. Conclusions

Functional studies reveal that AKR1B10 physiologically contributes to the maintenance of cellular homeostasis through the detoxification of cytotoxic RCS and regulating the metabolism of isoprenoid, retinoid, and lipid. AKR1B10 expression is constitutively high in the epithelial cells of the gastrointestinal tract, where the enzyme plays a critical role in epithelial cell renewal and the metabolism of several therapeutic drugs and carbonyl compounds produced by gut bacteria.

Clinical and basic research has proven that AKR1B10 is down-regulated in gastrointestinal cancers and several inflammatory bowel diseases, proposing that its low expression is correlated with poor prognosis and decreased survival for patients with CRC and gastric cancer. By contrast, in other tissues with normally low or no expression, the evidence is mounting that AKR1B10 is upregulated and involved in several forms of cancer and inflammatory disease. In addition, AKR1B10 is increased and implicated in the acquisition of anti-cancer drug resistance. In liver, lung, and breast cancers, AKR1B10 may be upregulated through the activation of stress-induced signal transduction pathways (such as Nrf2, AP-1, and MAPK pathways) and related to the proliferation and migration of cancer cells. However, the mechanisms underlying AKR1B10 down-regulation and its association with gastrointestinal diseases have been poorly elucidated. Thus, AKR1B10 acts as a double-edged sword depending on the type of cancer cells, and further understanding its role in the onset and progression of these diseases should be pursued.

A number of AKR1B10 inhibitors have been developed, of which HCCFA, the most potent, significantly inhibits not only the migration, proliferation, and metastasis of lung cancer A549 cells, but also the metastatic and invasive abilities of cisplatin-resistant A549 cells. Therefore, utilizing AKR1B10 inhibitors for cancers that highly express AKR1B10 is expected in clinical practice. Since no genes encoding homologues of human AKR1B10 were identified in experimental animals such as rats and mice [169,170], the efficacy and pharmacokinetics of the AKR1B10 inhibitors in vivo are unclear, and as such, further studies are needed to develop a more clinically relevant approach.

## Figures and Tables

**Table 1:** Agents that modulate AKR1B10 expression.

Agent *	Signal Molecule	Cell **	References
Up-regulation			
Ethoxyquin	Nrf2	Lung cancer A549, H23	[14]
MG-132, bortezomib	Nrf2	CRC SW-480, HT29	[17]
Doxorubicin	Nrf2	Gastric cancer MKN45	[18]
EGF, insulin	AP-1, ERK	HCC HepG2, Hep3B	[19]
Lipopolysaccharide		Blood mononuclear cells	[20]
BMP, IBMX		Mesenchymal stem cells	[21]
9,10-Phenanthrenequinone	Nrf2, ERK	Lung cancer A549	[22]
Cigarette smoke extract		Airway epithelium	[23]
Carnosic acid, *t*-BHQ	Nrf2	Astrocytoma U373MG	[15]
5-FU, L-OHP	p53	CRC HT116	[16]
Down-regulation			
TPA	c-Jun, ERK	Lung cancer A549	[15]
5-FU, L-OHP		CRC HT29	[16]

Note: * Abbreviations of agents: EGF; epidermal growth factor, BMP, bone morphogenetic protein 2; IBMX, 3-isobutyl-1-methylxanthine; *t*-BHQ, *t*-butyl hydroquinone; 5-FU, 5-fluorouracil; L-OHP, oxaliplatin; TPA, 12-*O*-tetradecanoylphorbol 13-acetate. ** Abbreviations of cancer: CRC, colorectal cancer; HCC, hepatocellular cancer.

**Table 2:** Kinetic constants for 4-hydroxynonenal of human AKRs and ALDHs.

Enzyme	*K*_m_ (μM)	*k*_cat_ (min^−1^)	*k*_cat_/*K*_m_ (min^−1^ μM^−1^)	Assay Condition (pH and °C)	References
AKR1B10	30.9	121	3.9	pH 7.0, 35 °C	[45]
	31	119	3.8	pH 7.0, 35 °C	[47]
	4.7	27	6.1	pH 7.4, 25 °C	[48]
AKR1B1	22	102	4.6	pH 7.0, 25 °C	[49]
	31	16.6	1.2	pH 7.0, 25 °C	[50]
	716	50	0.07	pH 6.4, 0.3 M Li_2_SO_4_, 35 °C	[47]
AKR1B15	2.2	5.2	2.5	pH 7.0, 25 °C	[5]
AKR1C1	34	8.8	0.27	pH 7.0, 37 °C	[51]
ALDH1A1	27	10	0.38	pH 8.0, 25 °C	[40]
ALDH1A2	7.5	31	4.1	pH 8.0, 25 °C	[40]
ALDH1A3	40	7.4	0.185	pH 8.0, 25 °C	[40]

**Table 3:** AKR1B10 expression in extra-hepatic cancers and its prognostic impact.

Organ	Cancer Type *	Assay	AKR1B10 Level **		Prognosis	References
			Lesion	Serum		
Colorectum	CRC, ADC	mRNA, protein	Low		Poor	[68,70,71,72,73,74,75,76]
		mRNA		Low	Poor	[24]
Stomach	ADC	mRNA, protein	Low		Poor	[69,77,78,79]
Lung	NSCLC, SCC, ADC	mRNA, protein	High		Poor	[8,81,82,83,84,85,86,87,88,89,90]
		protein		High		[90]
Breast	ADC, ductal carcinoma	mRNA, protein	High		Poor	[91,92,93,94,95,96]
		protein		High	Poor	[91]
Pancreas	ADC, MCT	protein	High			[55,97]
Oral cavity	SCC	protein	High		Poor	[98,99]

Note: * Abbreviations: ADC, adenocarcinoma; NSCLC, non-small cell lung carcinoma; SCC, squamous cell carcinoma; MCT, mucinous cystic tumor. ** Levels of cancerous lesions and sera of cancer patients compared to those of noncancerous lesions and sera of healthy doners.

**Table 4:** AKR1B10-upregulated chemoresistant cancer cells.

Cell		Drug *	Suggested Role of AKR1B10	References
CRC	HT29 cell	L-OHP	Promotion of cell proliferation by modulating isoprenoid metabolism.	[55]
	HT29 cell	MMC	Detoxification of RCS and drug metabolism	[143]
	Lovo cell	CDDP	Detoxification of RCS and down-regulation of PPARγ	[103]
	Lovo cell	DOX	Autophagy suppression by detoxification of RCS	[144]
Gastric cancer MKN45 cell		CDDP	Detoxification of RCS and down-regulation of PPARγ	[103]
		DOX	Detoxification of RCS and elevation of migrating and invasive potentials through MMP2 induction	[18]
Lung cancer A549 cell		CDDP	NO production by detoxification of RCS	[48]
		DTX	Detoxification of RCS	[145]
Breast cancer MCF-7 cell		DOX	Drug metabolism	[146]
Prostate cancer Du145 cell		DTX	Detoxification of RCS	[145]
Medulloblastoma D341 MED cell		CPA	Metabolism of a reactive metabolite, aldophosphamide	[147]

Note: * Abbreviations: L-OHP, oxaliplatin; MMC, mitomycin c; CDDP, cisplatin; DOX, doxorubicin; DTX, docetaxel; CPA, cyclophosphamide.

**Table 5:** Recently identified inhibitors of AKR1B10.

Inhibitor *	IC_50_ (µM) **		IC_50_ Ratio (1B10/1B1)	References
	AKR1B10	AKR1B1		
HCCFA	0.0035	0.277	79	[164]
HAHE	0.0062	4.9	790	[154]
MK204	0.080	21.7	271	[165]
Oleanolic acid	0.090	124	1370	[155]
Epalrestat	0.33	0.021	0.06	[156]
Androst-3β,5α,6β,19-tetrol	0.83	>100	120	[166]
Emodin	0.99	12	12	[157]
Arachidonic acid	1.1	24	22	[158]
Cohumulone	1.35	>125	>93	[159]
8-Prenylnaringenin	3.96	1.87	0.47	[160]
Glycyrrhetinic acid	4.9	280	57	[156]

Note: * Abbreviations: HCCFA, 7-hydroxy-2-oxo-2*H*-chromene-3-carboxylic acid [3-(4-fluorophenyl)propyl]amide; HAHE, 3-(4-hydroxy-2-methoxyphenyl)acrylic acid 3-(3-hydroxyphenyl)propyl ester; MK204, {5-chloro-2-[(pentabromobenzyl)carbamoyl]-phenoxy}acetic acid. ** IC_50_ value is determined with recombinant enzymes.
